# Supplementary material for: Single-cell and spatial transcriptomics analysis of non-small cell lung cancer
Source: Nat Commun. 2024 May 23;15:4388. doi: 10.1038/s41467-024-48700-8 (PMC11116453; doi:10.1038/s41467-024-48700-8)
Supplement: Supplementary file 1 — Supplementary Information [file 41467_2024_48700_MOESM1_ESM.pdf]

Supplementary Information

De Zuani et al.: Single-cell and spatial transcriptomics analysis of non-small cell lung cancer

Including:

Supplementary Notes

Supplementary Figure 1: Sorting strategy and scRNA-seq quality control

Supplementary Figure 2: Detailed annotation of the scRNA-seq dataset

Supplementary Figure 3: scRNA-seq metrics for detailed annotations

Supplementary Figure 4: LUAD and LUSC have similar cellular composition

Supplementary Figure 5: Detailed CNA results for each patient

Supplementary Figure 6: Foetal, tumour and background data integration

Supplementary Data description

Supplementary References

Supplementary Notes

Myeloid cells

We identified 214,960 and 160,921 myeloid cells in the tumour and B/H respectively, characterised by the global expression of *LYZ* and *SPI1*. We first identified in both data-sets a population of monocytes (expressing *CD14, IL1B*, *FCN1, CXCL8, S100A8,* and *S100A9*) and a broad population of macrophages (expressing *CD14*, *CD163, CD68, MARCO* and *MRC1*) (Figure 1 D, Supp. figure 2). From here we subclustered Mϕ to anti-inflammatory Mϕ (expressing *C1QB*, *C1QC*, *APOE*, *MRC1*, *MARCO*) and alveolar Mϕ (expressing *FABP4*, *MCEMP1* and *PPARG*). We mainly detected alveolar Mϕ in the B/H samples whereas the tumour samples were significantly enriched in monocyte-derived anti-inflammatory Mϕ, as previously described[1]. Anti-inflammatory Mϕ are involved in the resolution of inflammation and suppress the immunity against tumour cells, thus enabling the tumour microenvironment to promote cancer progression. We also noted an enhanced heterogeneity of anti-inflammatory Mϕ in the tumour, with several additional Mϕ states being uniquely present in tumour samples. Namely, we observed a population of cancer-associated macrophage-like cells (CAMLs) found to co-express epithelial (*KRT18*, *KRT19*, *EPCAM)* and macrophage markers (*LYZ*, *SPI2*, *CD14*, *CD163*, *C1QC*, *APOE*). Additionally, *STAB1*+ Mϕ, identified by high expression of scavenger receptor stabilin1 (*STAB1*), were also located in the tumour environment. High expression of *STAB1 in* Mϕ has been associated with poor prognosis in advanced cancers[2]. Finally, a population of cycling anti-inflammatory Mϕ was exclusively identified in tumour samples, characterised by the expression of *MKI67* and *TOP2A*.

Dendritic cells (DC) are professional antigen-presenting cells that were characterised by expression of *CLEC10A*, *CLEC4A*, and *CD1C*. Further subclustering of DCs revealed monocyte-derived DC2 (mo-DC2, expressing *CD14*, *CD163*, *MRC1*)[3] and conventional DC2 (cDC2) that lacked CD14 expression. Mo-DCs were more abundant in the tumour samples compared to background, consistent with an increased monocyte infiltration and differentiation. In B/H samples, populations of cycling mo-DC2 and cycling cDC2 cells were also identified (expressing *MKI67* and *TOP2A*).

Clusters of cells that had high expression of *LYZ and SPI1* and no expression of DC or monocyte/macrophage markers (e.g. *CD14* and *CD163,* respectively*)* were defined as immature myeloid cells.

T cells

T lymphocytes play a central role in engaging the immune system in fighting cancer. We collected 124,459 and 105,127 T cells in the tumour and B/H samples respectively. T cell populations (expressing *TRAC*, *CD3D*, *CD3E*, *CD3G)* across both data-sets included non-cytotoxic, cytotoxic T cells, naive T cells (expressing *CD40LG)* and downregulated T cells, corresponding to states with low or no expression of genes defining the exhausted, naive, or cytotoxic state, but with moderate expression of the global *CD3* markers. The non-cytotoxic state includes moderate expression of *CD4* and these cells play an active role in the adaptive immune system through the release of specific cytokines to control and polarise the immune response. In contrast, the cytotoxic T cells are programmed to recognise specific antigens and destroy pathogens. We identified two populations of cytotoxic T cells that displayed a graded expression of the cytotoxicity markers *GZMA*, *GZMB*, *GZMK*, *IFNG*, as well as *NKG7* and *PRF1*[4].

Across these cytotoxic and non-cytotoxic states we were also able to assess the degree of T cell exhaustion. In the tumour sample, we identified two populations of exhausted T cells, again with the varying degree of expression of cytotoxic genes leading us to classify these as exhausted cytotoxic T cells and exhausted T cells respectively. This exhausted state (expressing *TIGIT*, *CTLA4, PDCD1, LAG3*), indicates a reduced capacity to excrete cytokines, inhibiting the growth and activity of other immune cells. We further observed the tumour-specific exhausted states expressing *CXCL13*. Complementary studies[5] have indicated that immunosuppressive environments can lead to upregulation of *CXCL13* in T cell populations, consistent with our observation of *CXCL13* upregulation in the exhausted T cell clusters of

the tumour samples. We also identified a cycling state of exhausted cytotoxic T cells (additional expression of *MKI67* and *TOP2A*) in both samples.

Specifically in tumours we observed the regulatory T-cells (Tregs, expressing *FOXP3, CTLA4*, *TNFRSF18*)[6]. *FOXP3* is a transcription factor essential for the development and inhibitory function of Tregs. The suppressive activity of Treg involves the coordinate activation of *CTLA4* and *TNFRSF18* and repression of *IL2* and *IFNG*. Finally, in the B/H populations we identified a modest population of 5539 $\gamma\delta$ T cells, a rare population of unconventional T cells expressing the *CD8*+ and cytotoxic markers, as well as *GNLY*, *TRDC*, and *TRGC1*[7].

NK cells

We identified 36,046 and 80,703 NK cells in the tumour and B/H environments respectively, where in both samples two distinct populations of NK cells with high and low cytotoxicity were identified according to clear differences in the expression of *NKG7*, *PRF1*, *GZMA*, *GZMB*, *GZMM*, *GZMK*, *KLRB1*. In the B/H sample the higher cytotoxic state represented the majority of NK cells (71,833 out of 80,703 cells), while in tumour samples there was an even distribution of higher and lower cytotoxic NK cells. Consistent with this expansion of less cytotoxic NK cells, it was recently described that tumour cells are able to reprogram NKs inducing a resting phenotype which, in turn, promotes tumour metastases[8].

B cells

We identified 84,232 and 7,916 B cells in the tumour and B/H samples respectively. Common to both samples, mature B cells were defined based on their expression of global *CD79A, CD79B*, and *IGHM* markers. Varying expression of *JCHAIN*, *IGHG1*, *IGKC*, *IGHA1*, *XBP1*, and *MZB1* was used to identify plasma B and immature plasma B cells, whereas cycling plasma B cell showed substantial upregulation of *MKI67* and *TOP2A*. Unique to the tumour sample, we also identified a population of LYZ+ B cells, with upregulated *LYZ* expression common to the myeloid cell states, a set of Downregulated B cells which downregulated most of B cells markers (e.g. *CD79A*, *CD79B*, *MS4A1*) and TNF+ B cells that co-expressed B cell and T cell specific genes (*MS4A1*, *CD79A*, *CD3D*, *CD3E*, *CD3G*) in addition to a high level of *TNF*.

In B/H we also identified a small population of 526 NKB cells, identified through the shared expression of global NK (*NKG7*, *PRF1*) and B cell (*IGHM, CD19, and MS4A1*) markers[9], in addition to *ID2* (a key transcription factor for NK development). NKB cells are reported to have unique immunity features which distinguish them from B and T cells, including the specific excretion of IL12 and IL18 as a means of eradicating microbial infection[10].

Mast cells

We identified two distinct populations of mast cells in both the tumour and B/H samples (5,255 cells and 4,387 cells respectively). Mast cells were identified through the high expression of *HDC*, as well as *MITF* and *GATA2*, key transcription factors regulating mast cell identity and responsiveness to external factors such as antigenic stimulation[11]. The high expression of *MKI67* and *TOP2A* also allowed us to resolve a second population of cycling mast cells.

Non-immune cells

The non-immune component of the TME has been implicated in resistance to multiple types of cancer therapy. In tumour and B/H data-sets we identified 36,025 and 23,572 epithelial cells respectively (expressing *EPCAM*, *CDH1*, *KRT18*, and *KRT19*) that were further subclustered to ciliated cells (expressing *FOXJ1*, and *RFX2*), alveolar type 2 (AT2) cells (expressing *SFTPB*, *MUC1*, *SFTPC*), cycling AT2 cells (additionally expressing *MKI67* and *TOP2A*) and two distinct clusters of epithelial cells present in tumours, namely atypical epithelial cells which downregulated epithelial markers and transitioning epithelial cells which upregulated myeloid markers. In B/H, but not in tumour, we identified a population of club cells (expressing *SCGB1A1*). A separate population of cycling epithelial cells (based on high *MKI67*

expression) were identified in the tumour, and annotated as a distinct phenotype based on their separation in UMAP-space.

Common to both tumour and B/H we identified respectively 304 and 4,105 lymphatic endothelial cells (expressing endothelial markers *CLDN5, PECAM1* as well as lymphatic endothelium associated genes *RAMP2*, *CCL21*, *LYVE1*). Next, we identified fibroblasts (high expression of *COL1A1*, *COL1A2*, *SPARC*, and *DCN*) as well as activated adventitial fibroblasts (co-expressing *SERPINF1* and *THY1* and α smooth muscle actin, *ACTA2*).

Consistent with a recent report[12], we did not identify AT1 cells in either tumour or B/H tissues.

Evaluating cell-type annotations following label transfer

To test consistency in cell-type annotation performed separately in tumour and B/H, we performed reference-query mapping from tumour to B/H using scArches[13] (see Methods). The cell type proportions obtained by scVI integration across samples and label transfer correlated closely with separate dataset proportions (Pearson correlation coefficient 0.89). However, we observed discrepancy between the relative abundance of DCs and monocytes. Considering that monocytes can differentiate to DCs under inflammatory conditions these two populations can have similar transcriptional signatures. To test whether monocytes (from separate annotations) assigned to DCs through label transfer express putative DC markers, we plotted a set of known monocyte and DC genes and examined their expression across relevant populations. Our analysis showed that monocytes assigned to be DCs following label transfer do not express putative DC genes such as *CDC1*, *CLEC4A*, *CLEC10A* confirming the correctness of separate annotations (Suppl Figure 2A-C).

Supplementary Figures

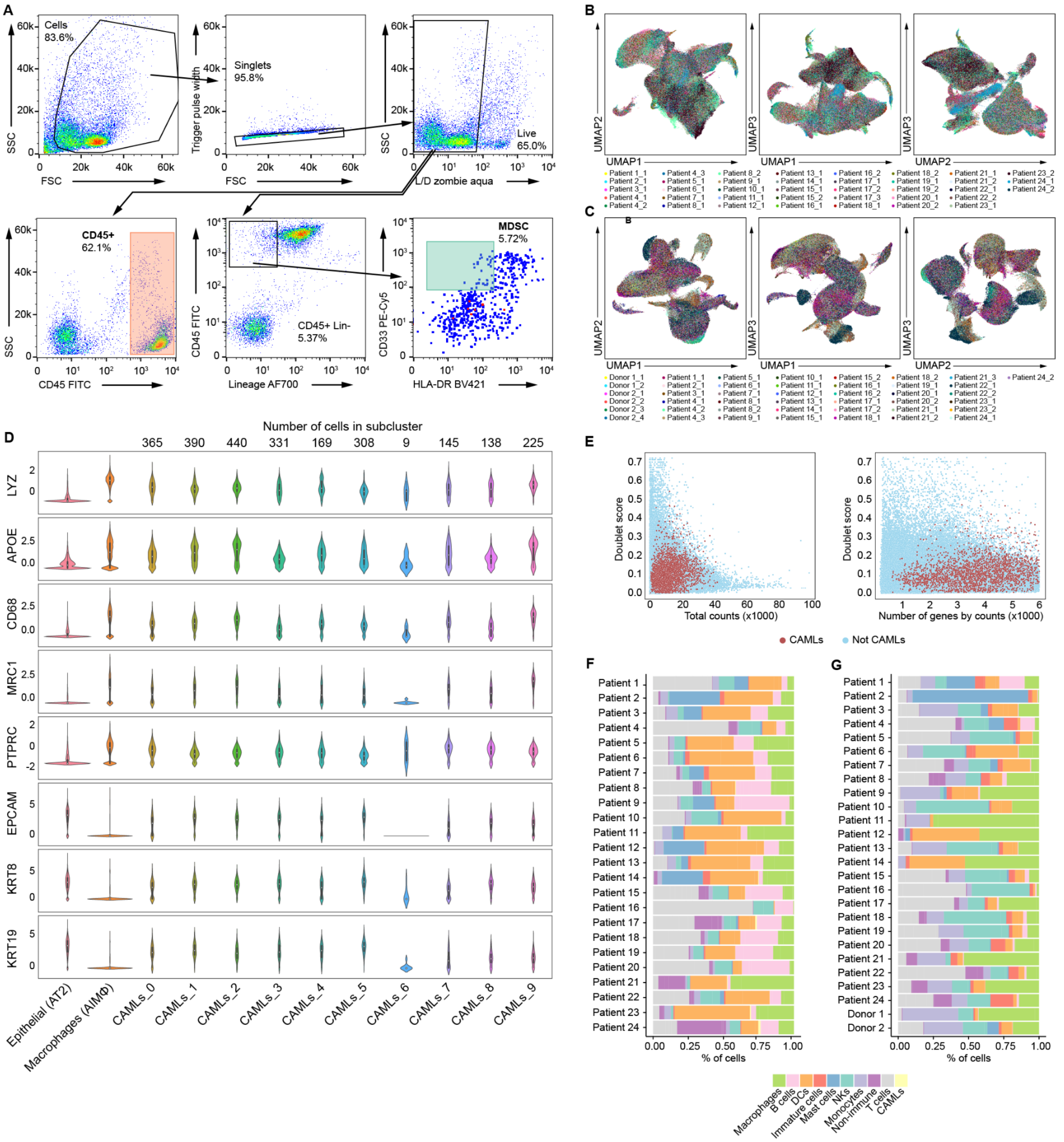

Supplementary Figure 1: Sorting strategy and scRNA-seq quality control

A. Representative plots showing the gating strategy used to enrich CD45+ and MDSCs. Debris was excluded by gating on FSC-A/SSC-A; singlets were gated on FCS/TPW; dead cells were excluded by

gating on Zombie-aqua negative cells, then CD45+ cells were sorted. MDSCs were identified as CD45 positive, Lineage (CD3, CD19, CD56) negative, CD33 positive, HLA-DR dim/negative cells.

B and C. UMAP projection of the tumour (**B**) and combined B/H (**C**) datasets after batch correction. Colours represent each library sequenced.

D. Violin plot showing the scaled, normalised, log-transformed gene expression of myeloid- (*LYZ, APOE, CD68, MRC1*), immune- (*PTPRC*) and epithelial- (*EPCAM, KRT8, KRT19*) specific genes in AT2 cells, anti-inflammatory macrophages and 10 CAML subclusters calculated by k-means. The values above each plot indicate the number of cells in each sub-cluster.

E. Scatterplot showing the doublet score estimated by Scrublet for each individual cell (y axis) versus total counts (x axis - left) and number of genes by counts (x axis - right). CAMLs are highlighted in brown, all other cell types are in light blue.

F and G. Relative proportion of broad immune and non-immune cells in the tumour (**F**) and background and healthy (**G**) datasets, calculated within all libraries in each patient.

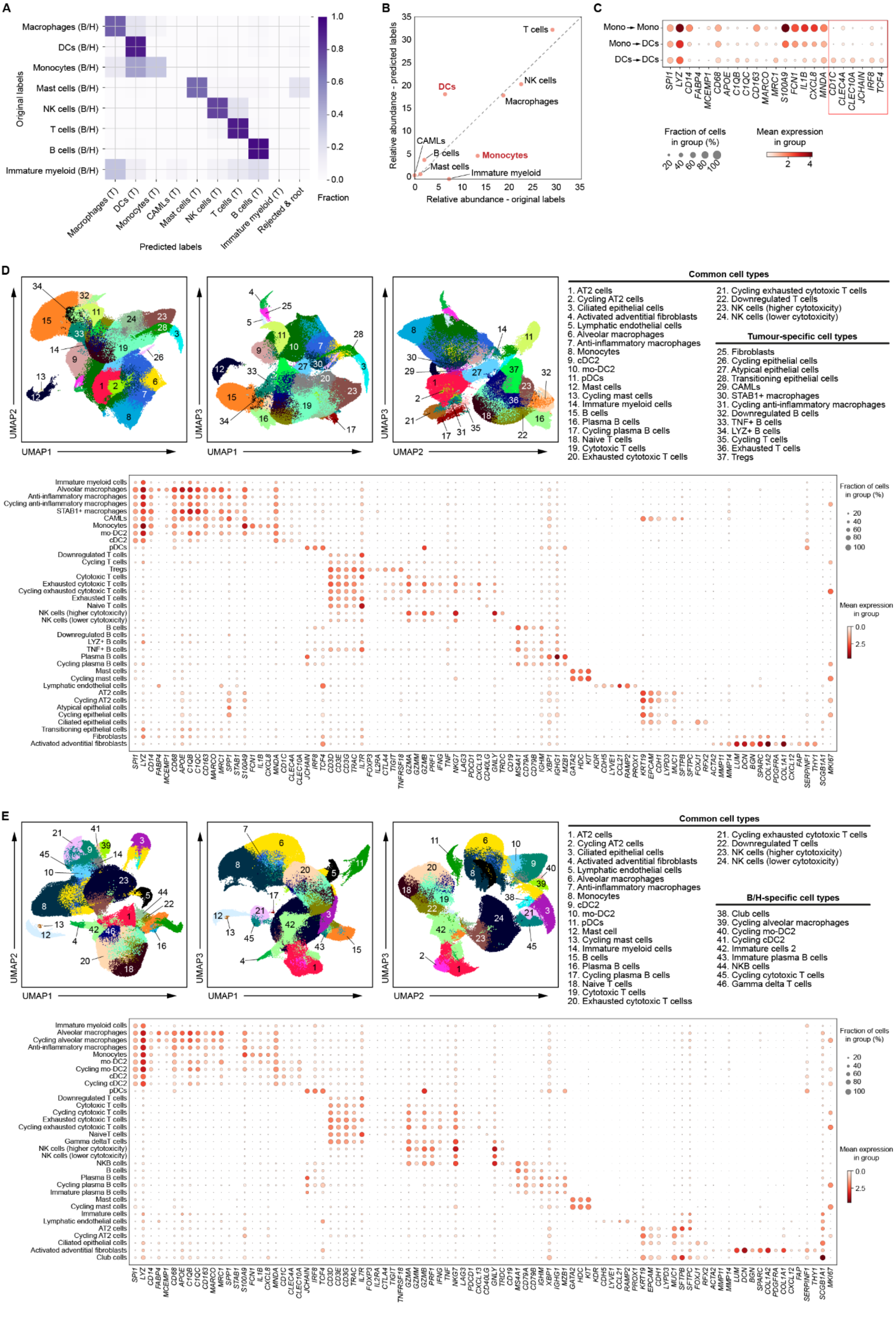

A
Macrophages (B/H)
DCs (B/H)
Monocytes (B/H)
Mast cells (B/H)
NK cells (B/H)
T cells (B/H)
B cells (B/H)
Immature myeloid (B/H)
Original labels
Macrophages (T)
DCs (T)
Monocytes (T)
CAMLs (T)
Mast cells (T)
NK cells (T)
T cells (T)
B cells (T)
Immature myeloid (T)
Rejected & root
Predicted labels
Fraction
1.0
0.8
0.6
0.4
0.2
0.0
B
Relative abundance - predicted labels
Relative abundance - original labels
T cells
DCs
NK cells
Macrophages
CAMLs
B cells
Monocytes
Mast cells
Immature myeloid
C
Mono → Mono
Mono → DCs
DCs → DCs
SPI1 LYZ CD14 FABP4 MCEMP1 CD68 APOE C1QB C1QC CD163 MARCO MRC1 S100A9 FCN1 IL1B CXCL8 MNDA CD1C CLEC4A CLEC10A JCHAIN IRF8 TCF4
Fraction of cells in group (%)
20 40 60 80 100
Mean expression in group
2 4
D
UMAP1
UMAP2
UMAP3
Common cell types
1. AT2 cells
2. Cycling AT2 cells
3. Ciliated epithelial cells
4. Activated adventitial fibroblasts
5. Lymphatic endothelial cells
6. Alveolar macrophages
7. Anti-inflammatory macrophages
8. Monocytes
9. cDC2
10. mo-DC2
11. pDCs
12. Mast cells
13. Cycling mast cells
14. Immature myeloid cells
15. B cells
16. Plasma B cells
17. Cycling plasma B cells
18. Naive T cells
19. Cytotoxic T cells
20. Exhausted cytotoxic T cells
21. Cycling exhausted cytotoxic T cells
22. Downregulated T cells
23. NK cells (higher cytotoxicity)
24. NK cells (lower cytotoxicity)
Tumour-specific cell types
25. Fibroblasts
26. Cycling epithelial cells
27. Atypical epithelial cells
28. Transitioning epithelial cells
29. CAMLs
30. STAB1+ macrophages
31. Cycling anti-inflammatory macrophages
32. Downregulated B cells
33. TNF+ B cells
34. LYZ+ B cells
35. Cycling T cells
36. Exhausted T cells
37. Tregs
Immature myeloid cells
Alveolar macrophages
Anti-inflammatory macrophages
Cycling anti-inflammatory macrophages
STAB1+ macrophages
CAMLs
Monocytes
mo-DC2
cDC2
pDCs
Downregulated T cells
Cycling T cells
Tregs
Cytotoxic T cells
Exhausted cytotoxic T cells
Cycling exhausted cytotoxic T cells
Exhausted T cells
Naive T cells
NK cells (higher cytotoxicity)
NK cells (lower cytotoxicity)
B cells
Downregulated B cells
LYZ+ B cells
TNF+ B cells
Plasma B cells
Cycling plasma B cells
Mast cells
Cycling mast cells
Lymphatic endothelial cells
AT2 cells
Cycling AT2 cells
Atypical epithelial cells
Cycling epithelial cells
Ciliated epithelial cells
Transitioning epithelial cells
Fibroblasts
Activated adventitial fibroblasts
SPI1 LYZ CD14 FABP4 MCEMP1 CD68 APOE C1QB C1QC CD163 MARCO MRC1 SPP1 STAB1 S100A9 FCN1 IL1B CXCL8 MNDA CD1C CLEC4A CLEC10A JCHAIN IRF8 TCF4 CD3D CD3E CD3G TRAC IL7R FOXP3 IL2RA CTLA4 TIGIT TNFRSF18 GZMA GZMM GZMB PRF1 IFNG TNF NKG7 LAG3 PDCD1 CXCL13 CD40LG GNLY TRDC CD19 MS4A1 CD79A CD79B IGHM XBP1 IGHG1 MZB1 GATA2 HDC KIT KDR CDH5 LYVE1 CCL21 RAMP2 PROX1 KRT19 EPCAM CDH1 LYPD3 MUC1 SFTPB SFTPC FOXJ1 RFX2 ACTA2 MMP11 MMP14 LUM DCN BGN SPARC COL1A2 PDGFRA COL1A1 CXCL12 FAP SERPINF1 THY1 SCGB1A1 MKI67
Fraction of cells in group (%)
20
40
60
80
100
Mean expression in group
0.0
2.5
E
UMAP1
UMAP2
UMAP3
Common cell types
1. AT2 cells
2. Cycling AT2 cells
3. Ciliated epithelial cells
4. Activated adventitial fibroblasts
5. Lymphatic endothelial cells
6. Alveolar macrophages
7. Anti-inflammatory macrophages
8. Monocytes
9. cDC2
10. mo-DC2
11. pDCs
12. Mast cell
13. Cycling mast cells
14. Immature myeloid cells
15. B cells
16. Plasma B cells
17. Cycling plasma B cells
18. Naive T cells
19. Cytotoxic T cells
20. Exhausted cytotoxic T cellss
21. Cycling exhausted cytotoxic T cells
22. Downregulated T cells
23. NK cells (higher cytotoxicity)
24. NK cells (lower cytotoxicity)
B/H-specific cell types
38. Club cells
39. Cycling alveolar macrophages
40. Cycling mo-DC2
41. Cycling cDC2
42. Immature cells 2
43. Immature plasma B cells
44. NKB cells
45. Cycling cytotoxic T cells
46. Gamma delta T cells
Immature myeloid cells
Alveolar macrophages
Cycling alveolar macrophages
Anti-inflammatory macrophages
Monocytes
mo-DC2
Cycling mo-DC2
cDC2
Cycling cDC2
pDCs
Downregulated T cells
Cytotoxic T cells
Cycling cytotoxic T cells
Exhausted cytotoxic T cells
Cycling exhausted cytotoxic T cells
NaiveT cells
Gamma deltaT cells
NK cells (higher cytotoxicity)
NK cells (lower cytotoxicity)
NKB cells
B cells
Plasma B cells
Cycling plasma B cells
Immature plasma B cells
Mast cells
Cycling mast cells
Immature cells
Lymphatic endothelial cells
AT2 cells
Cycling AT2 cells
Ciliated epithelial cells
Activated adventitial fibroblasts
Club cells
SPI1 LYZ CD14 FABP4 MCEMP1 CD68 APOE C1QB C1QC CD163 MARCO MRC1 SPP1 STAB1 S100A9 FCN1 IL1B CXCL8 MNDA CD1C CLEC4A CLEC10A JCHAIN IRF8 TCF4 CD3D CD3E CD3G TRAC IL7R FOXP3 IL2RA CTLA4 TIGIT TNFRSF18 GZMA GZMM GZMB PRF1 IFNG TNF NKG7 LAG3 PDCD1 CXCL13 CD40LG GNLY TRDC CD19 MS4A1 CD79A CD79B IGHM XBP1 IGHG1 MZB1 GATA2 HDC KIT KDR CDH5 LYVE1 CCL21 RAMP2 PROX1 KRT19 EPCAM CDH1 LYPD3 MUC1 SFTPB SFTPC FOXJ1 RFX2 ACTA2 MMP11 MMP14 LUM DCN BGN SPARC COL1A2 PDGFRA COL1A1 CXCL12 FAP SERPINF1 THY1 SCGB1A1 MKI67
Fraction of cells in group (%)
20
40
60
80
100
Mean expression in group
0.0
2.5

Supplementary Figure 2: Detailed annotation of the scRNA-seq dataset

A. Heatmap representing the results of label transfer. Each square represents the fraction of cells in each broad category on the B/H dataset (original labels) that was assigned to each tumour label (predicted labels).

B. Scatterplot representing the relative abundance of each broad immune cell in the B/H dataset before (original labels) and after label transfer (predicted labels).

C. Dotplot showing the expression of myeloid marker genes in the B/H dataset for monocytes that were labelled as monocytes (mono→mono), monocytes that were labelled as DCs (mono→DCs), and DCs that were labelled as DCs (DCs→DCs). The size of each dot represents the percentage of cells in the cluster expressing the gene, while the colour represents the mean normalised scaled log-transformed expression of each gene in each cluster.

D. UMAP projection (top) and dotplot (bottom) of representative genes used for detailed cell type annotations in the tumour dataset. Colours and numbers represent detailed cell type annotations. The size of each dot represents the percentage of cells in the cluster expressing the gene, while the colour represents the mean normalised scaled log-transformed expression of each gene in each cluster.

E. UMAP projection (top) and dotplot (bottom) of representative genes used for detailed cell type annotations in the B/H dataset. Colours and numbers represent detailed cell type annotations. The size of each dot represents the percentage of cells in the cluster expressing the gene, while the colour represents the mean normalised scaled log-transformed expression of each gene in each cluster.

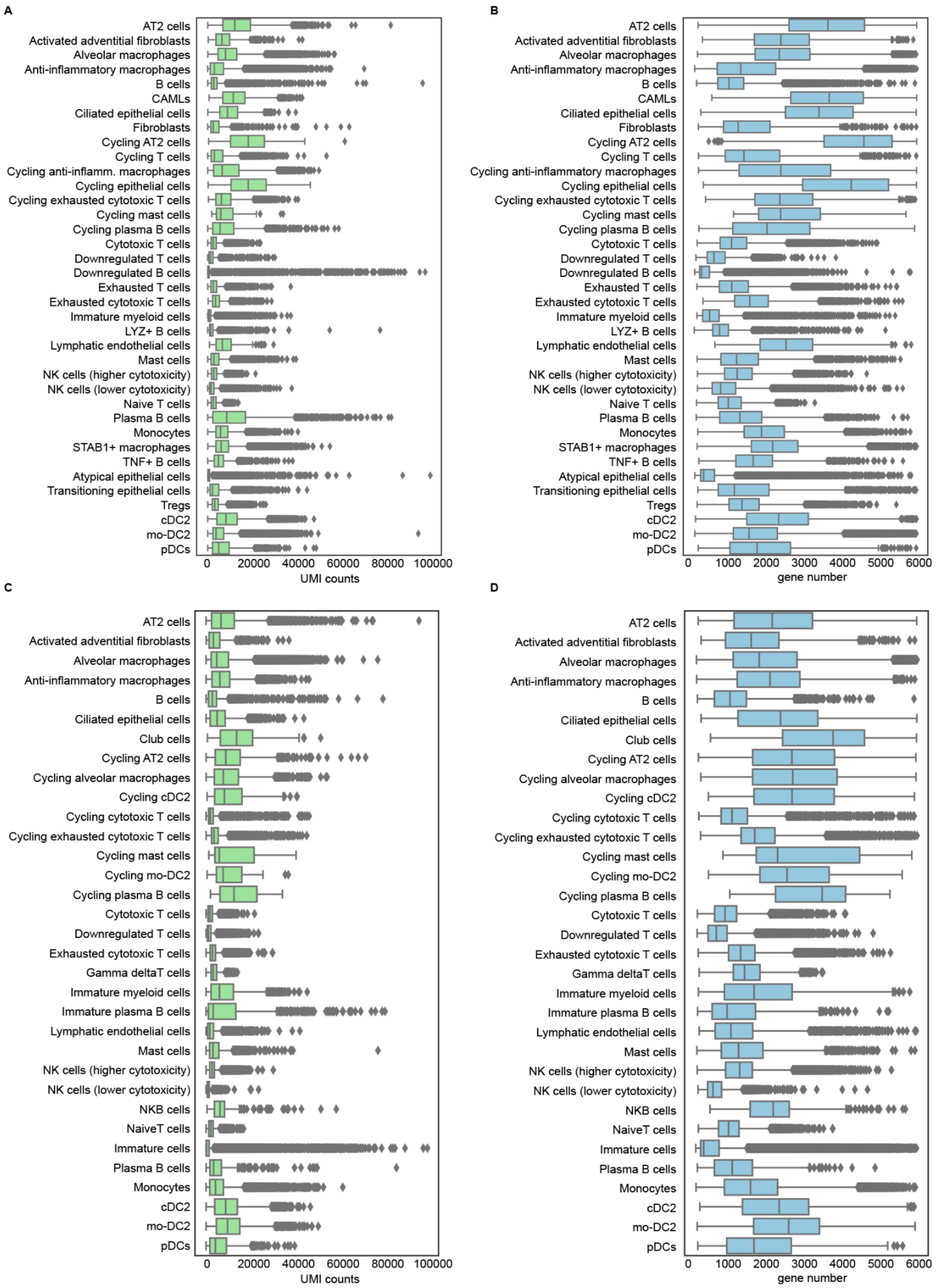

Supplementary Figure 3: scRNA-seq metrics for detailed annotations

A and B. Number of UMIs (**A**) and genes (**B**) per cell type in the tumour dataset.

C and D. Number of UMIs (**C**) and genes (**D**) per cell type in the B/H dataset.

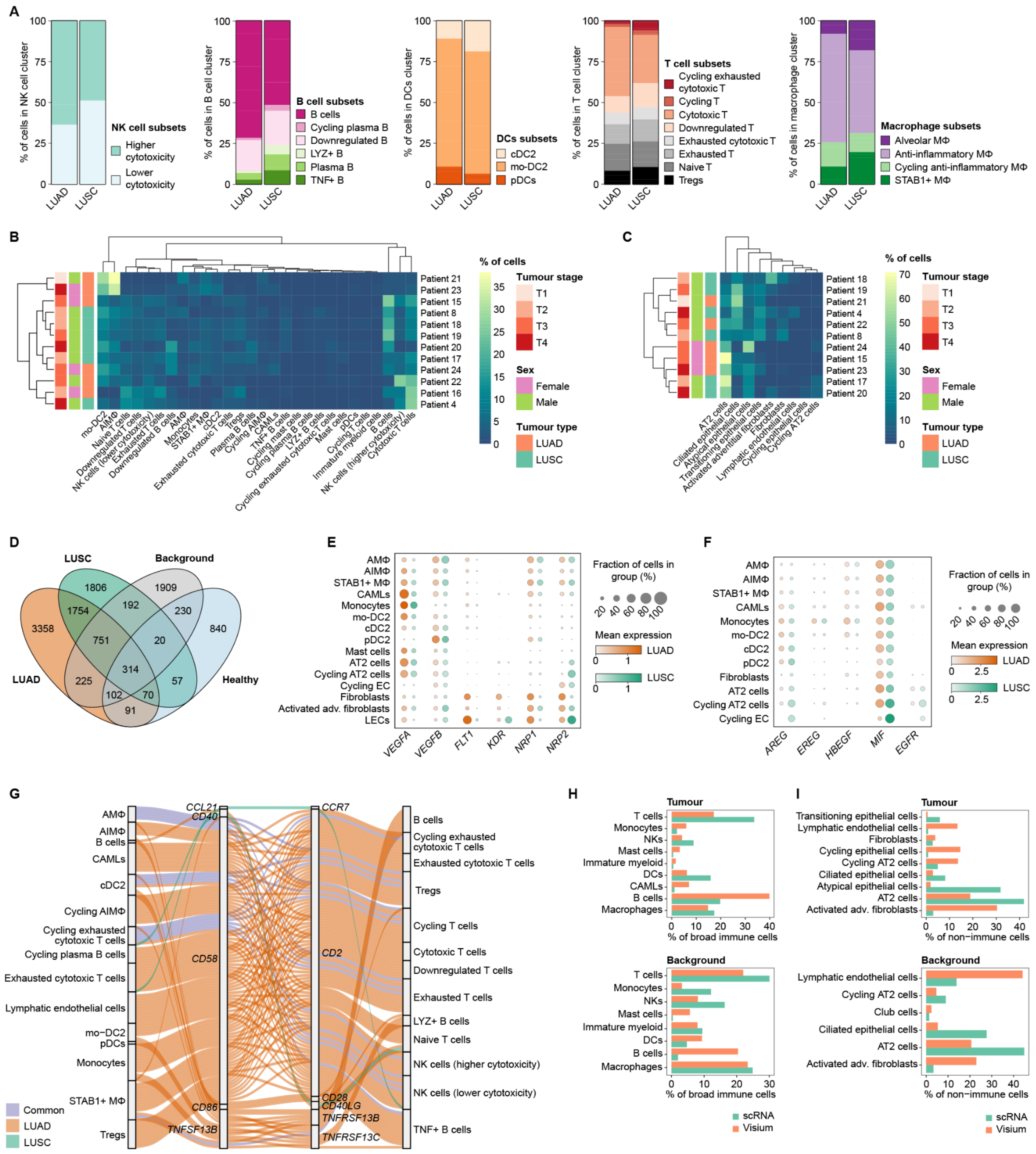
A
% of cells in NK cell cluster
LUAD
LUSC
NK cell subsets
Higher cytotoxicity
Lower cytotoxicity
% of cells in B cell cluster
B cell subsets
B cells
Cycling plasma B
Downregulated B
LYZ+ B
Plasma B
TNF+ B
% of cells in DCs cluster
DCs subsets
cDC2
mo-DC2
pDCs
% of cells in T cell cluster
T cell subsets
Cycling exhausted cytotoxic T
Cycling T
Cytotoxic T
Downregulated T
Exhausted cytotoxic T
Exhausted T
Naive T
Tregs
% of cells in macrophage cluster
Macrophage subsets
Alveolar MΦ
Anti-inflammatory MΦ
Cycling anti-inflammatory MΦ
STAB1+ MΦ
B
Patient 21
Patient 23
Patient 15
Patient 8
Patient 18
Patient 19
Patient 20
Patient 17
Patient 24
Patient 22
Patient 16
Patient 4
mo-DC2
AIMΦ
Naive T cells
Downregulated T cells
NK cells (lower cytotoxicity)
Exhausted T cells
Downregulated B cells
AMΦ
Monocytes
STAB1+ MΦ
cDC2
Exhausted cytotoxic T cells
Tregs
Plasma B cells
Cycling AIMΦ
CAMLs
TNF+ B cells
Cycling mast cells
Cycling plasma B cells
LYZ+ B cells
Cycling exhausted cytotoxic T cells
Mast cells
pDCs
Cycling T cells
Immature myeloid cells
B cells
NK cells (higher cytotoxicity)
Cytotoxic T cells
% of cells
Tumour stage
T1
T2
T3
T4
Sex
Female
Male
Tumour type
LUAD
LUSC
C
Patient 18
Patient 19
Patient 21
Patient 4
Patient 22
Patient 8
Patient 24
Patient 15
Patient 23
Patient 17
Patient 20
AT2 cells
Ciliated epithelial cells
Atypical epithelial cells
Transitioning epithelial cells
Activated adventitial fibroblasts
Fibroblasts
Lymphatic endothelial cells
Cycling epithelial cells
Cycling AT2 cells
D
LUSC
Background
LUAD
Healthy
1806
1909
1754
192
230
3358
751
20
840
314
225
57
102
70
91
E
AMΦ
AIMΦ
STAB1+ MΦ
CAMLs
Monocytes
mo-DC2
cDC2
pDC2
Mast cells
AT2 cells
Cycling AT2 cells
Cycling EC
Fibroblasts
Activated adv. fibroblasts
LECs
VEGFA
VEGFB
FLT1
KDR
NRP1
NRP2
Fraction of cells in group (%)
Mean expression
LUAD
LUSC
F
AMΦ
AIMΦ
STAB1+ MΦ
CAMLs
Monocytes
mo-DC2
cDC2
pDC2
Fibroblasts
AT2 cells
Cycling AT2 cells
Cycling EC
AREG
EREG
HBEGF
MIF
EGFR
Fraction of cells in group (%)
Mean expression
LUAD
LUSC
G
AMΦ
AIMΦ
B cells
CAMLs
cDC2
Cycling AIMΦ
Cycling exhausted cytotoxic T cells
Cycling plasma B cells
Exhausted cytotoxic T cells
Lymphatic endothelial cells
mo-DC2
pDCs
Monocytes
STAB1+ MΦ
Tregs
CCL21
CD40
CD58
CD86
TNFSF13B
CCR7
CD2
CD28
CD40LG
TNFRSF13B
TNFRSF13C
B cells
Cycling exhausted cytotoxic T cells
Exhausted cytotoxic T cells
Tregs
Cycling T cells
Cytotoxic T cells
Downregulated T cells
Exhausted T cells
LYZ+ B cells
Naive T cells
NK cells (higher cytotoxicity)
NK cells (lower cytotoxicity)
TNF+ B cells
Common
LUAD
LUSC
H
Tumour
T cells
Monocytes
NKs
Mast cells
Immature myeloid
DCs
CAMLs
B cells
Macrophages
% of broad immune cells
Background
T cells
Monocytes
NKs
Mast cells
Immature myeloid
DCs
B cells
Macrophages
% of broad immune cells
scRNA
Visium
I
Tumour
Transitioning epithelial cells
Lymphatic endothelial cells
Fibroblasts
Cycling epithelial cells
Cycling AT2 cells
Ciliated epithelial cells
Atypical epithelial cells
AT2 cells
Activated adv. fibroblasts
% of non−immune cells
Background
Lymphatic endothelial cells
Cycling AT2 cells
Club cells
Ciliated epithelial cells
AT2 cells
Activated adv. fibroblasts
% of non−immune cells
scRNA
Visium

Supplementary Figure 4: LUAD and LUSC have similar cellular composition

A. Relative proportion of NK, B, DC, T and macrophages subsets within the broad annotation in LUAD (left) and LUSC (right), calculated within the CD235- enrichment.

B and C. Heatmap showing the relative proportion of all immune cell subsets (**B**) and non-immune cell subsets (**C**) in each patient within the CD235- enrichment. Annotations indicate the broad tumour stage (T1-T4), cancer type (LUAD, LUSC and non-annotated lung cancer LC), and sex. Rows and columns were hierarchically clustered using the complete linkage method on euclidean distances.

D. Venn diagram showing the number of significant cell-cell L-R pairs identified by cellphoneDB independently on the tumour dataset split on LUAD and LUSC, the background dataset, and the healthy dataset.

E. Dotplot showing the expression of the genes highlighted in the Sankey diagram in Figure 2E.

F. Dotplot showing the expression of the genes highlighted in the Sankey diagram in Figure 2F.

G. Sankey diagram showing the tumour-specific interactions in LUAD and LUSC for co-stimulatory molecules detected by cellphoneDB. Line colour identifies whether the LR interaction between each cell type was found in LUAD only (orange), in LUSC only (green) or in both tumours (blue).

H. Barplot showing the relative abundance of broad immune cell types calculated from the scRNA-seq dataset (green) and from the VIsium dataset (orange).

I. Barplot showing the relative abundance of non-immune cell types calculated from the scRNA-seq dataset (green) and from the VIsium dataset (orange).

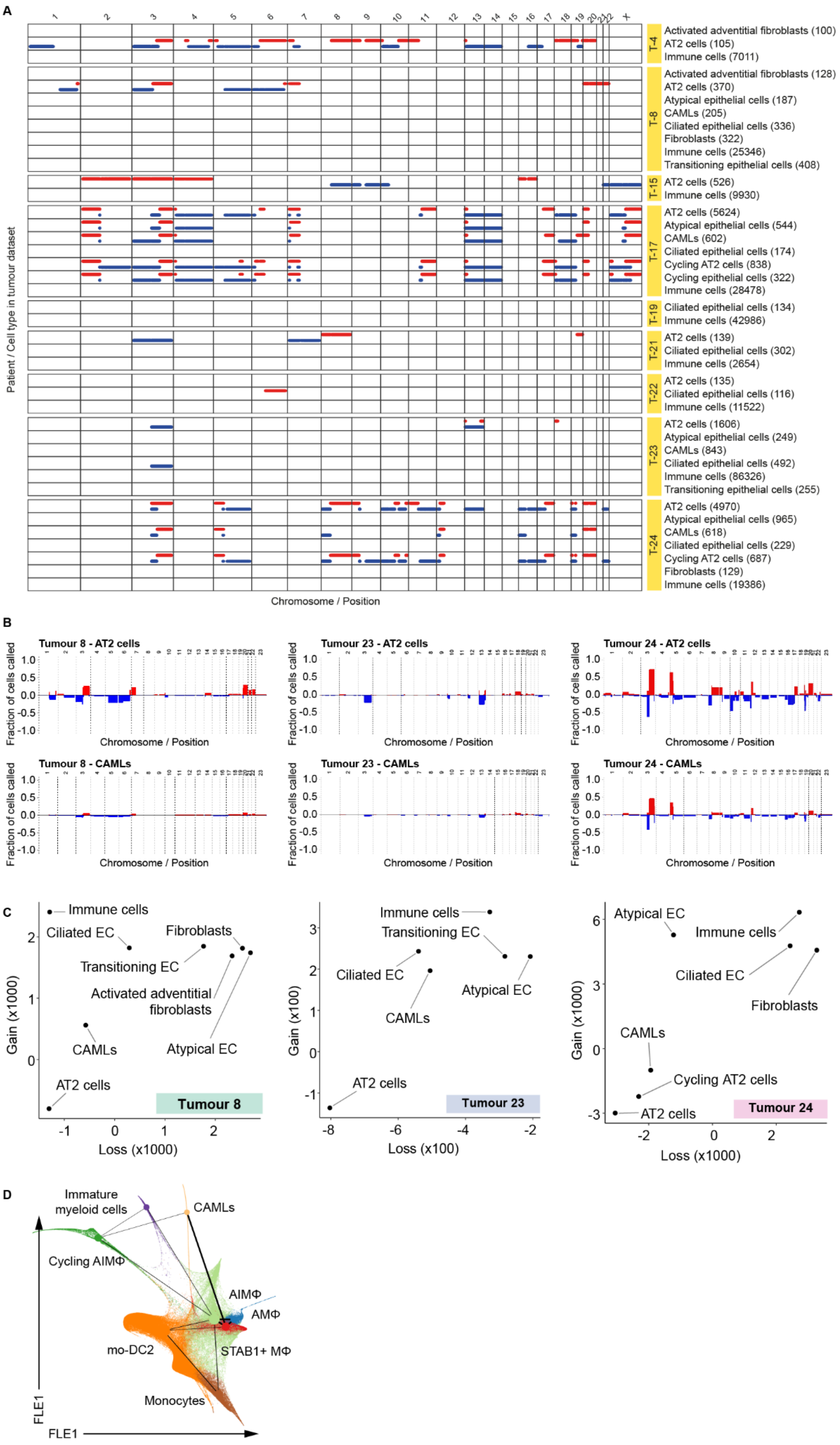

A
1 2 3 4 5 6 7 8 9 10 11 12 13 14 15 16 17 18 19 20 21 22 X
Patient / Cell type in tumour dataset
Chromosome / Position
T-4
Activated adventitial fibroblasts (100)
AT2 cells (105)
Immune cells (7011)
T-8
Activated adventitial fibroblasts (128)
AT2 cells (370)
Atypical epithelial cells (187)
CAMLs (205)
Ciliated epithelial cells (336)
Fibroblasts (322)
Immune cells (25346)
Transitioning epithelial cells (408)
T-15
AT2 cells (526)
Immune cells (9930)
T-17
AT2 cells (5624)
Atypical epithelial cells (544)
CAMLs (602)
Ciliated epithelial cells (174)
Cycling AT2 cells (838)
Cycling epithelial cells (322)
Immune cells (28478)
T-19
Ciliated epithelial cells (134)
Immune cells (42986)
T-21
AT2 cells (139)
Ciliated epithelial cells (302)
Immune cells (2654)
T-22
AT2 cells (135)
Ciliated epithelial cells (116)
Immune cells (11522)
T-23
AT2 cells (1606)
Atypical epithelial cells (249)
CAMLs (843)
Ciliated epithelial cells (492)
Immune cells (86326)
Transitioning epithelial cells (255)
T-24
AT2 cells (4970)
Atypical epithelial cells (965)
CAMLs (618)
Ciliated epithelial cells (229)
Cycling AT2 cells (687)
Fibroblasts (129)
Immune cells (19386)
B
Tumour 8 - AT2 cells
Tumour 23 - AT2 cells
Tumour 24 - AT2 cells
Tumour 8 - CAMLs
Tumour 23 - CAMLs
Tumour 24 - CAMLs
Fraction of cells called
Chromosome / Position
1.0 0.5 0.0 -0.5 -1.0
C
Immune cells
Ciliated EC
Fibroblasts
Transitioning EC
Activated adventitial fibroblasts
CAMLs
Atypical EC
AT2 cells
Tumour 8
Gain (x1000)
Loss (x1000)
Immune cells
Transitioning EC
Ciliated EC
Atypical EC
CAMLs
AT2 cells
Tumour 23
Gain (x100)
Loss (x100)
Atypical EC
Immune cells
Ciliated EC
Fibroblasts
CAMLs
Cycling AT2 cells
AT2 cells
Tumour 24
Gain (x1000)
Loss (x1000)
D
Immature myeloid cells
CAMLs
Cycling AIMΦ
AIMΦ
AMΦ
mo-DC2
STAB1+ MΦ
Monocytes
FLE1
FLE1

Supplementary Figure 5: Detailed CNA results for each patient

A. CNA analysis. The plot shows chromosomal gains (red lines) and losses (blue lines) estimated by CopyKat in each chromosome arm, for different cell types and all patients in the tumour dataset. All immune cell types were grouped together for plotting purposes.

B. Detailed overview of CNAs in AT2 and CAMLs from each patient where we detected CAMLs and AT2 cells. Bars indicate the frequency of cells harbouring chromosomal gains (red bar) or losses (blue bars) in specific chromosomal regions in the tumour dataset.

C. Scatterplot of the KL divergence of losses (x-axis) and gains (y-axis) between each cell type calculated using their gain and losses distribution for the same patient in panel B. All immune cell types were grouped together for plotting purposes.

D. PAGA graph overlaid on the diffusion maps (force-directed layout - FLE embedding) computed for macrophage clusters, immature myeloid cells and CAMLs in tumour.

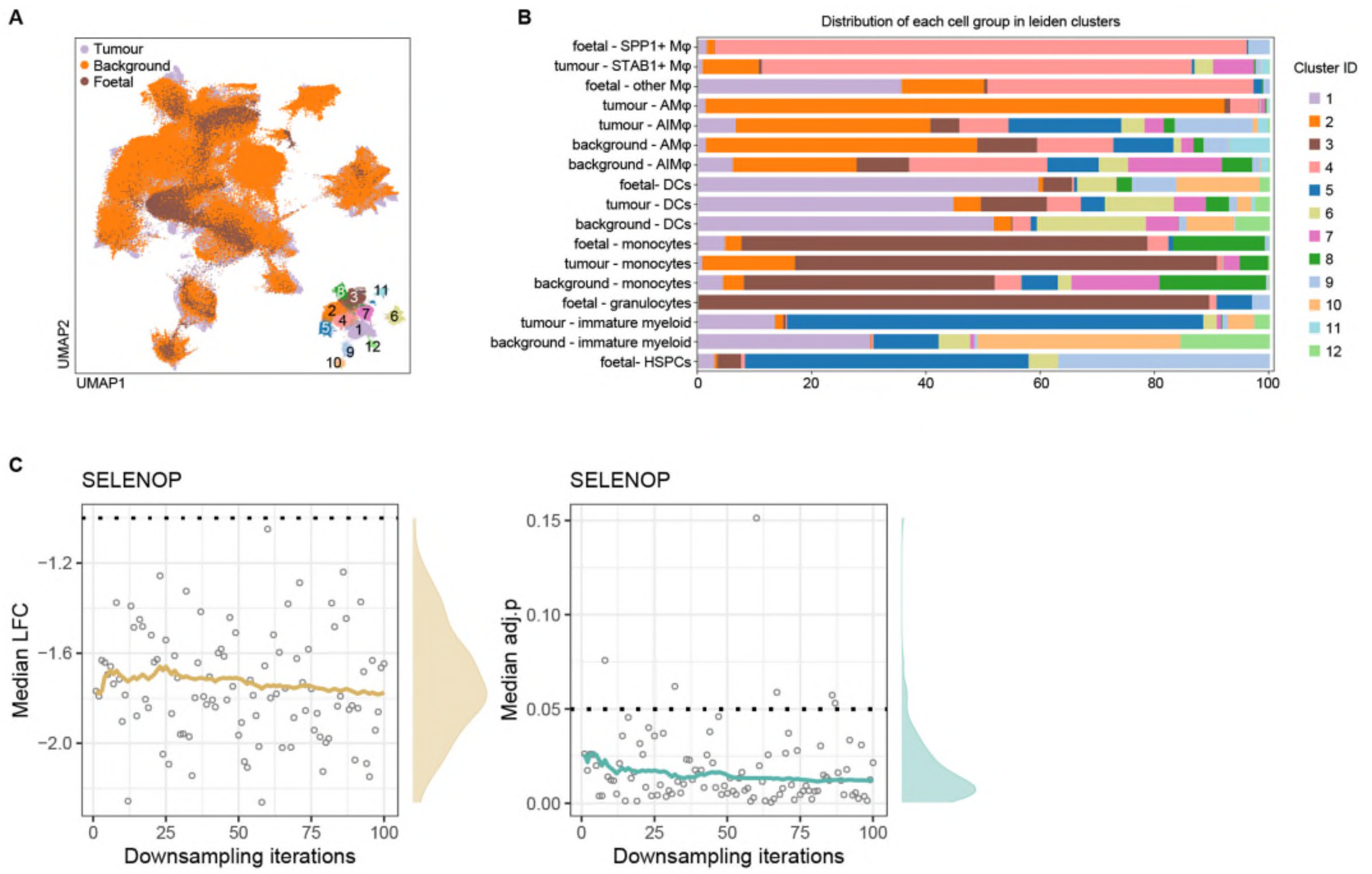

Supplementary Figure 6: Foetal, tumour and background data integration

A. UMAP of the integrated foetal lung myeloid, background myeloid and tumour myeloid dataset, coloured by the origin of each cell. In the inlet, same UMAP coloured by Leiden cluster.

B. Distribution of each cell group in the leiden clusters of the integrated foetal lung myeloid, background myeloid and tumour myeloid dataset.

C. Median log(fold-change) and adjusted p-value for each DEA iteration between AMϕ and STAB1+ Mϕ. Coloured lines depict the moving median value across 100 iterations.

Supplementary Data

Supplementary Data consists of one .xlsx file containing 22 tables:

Supplementary Data 1: Sample metadata, including scRNA-seq metrics, spatial transcriptomics metrics, and available clinical data.

Supplementary Data 2: Results of the Wilcoxon Rank Sum and Bonferroni correction on the non-immune cell type abundance in the unenriched cell samples (CD235-), comparing tumour and background.

Supplementary Data 3: Results of the Wilcoxon Rank Sum and Bonferroni correction on the broad immune cell type abundance in the unenriched cell samples (CD235-), comparing tumour and background.

Supplementary Data 4: Cell per cluster, per patient, for the tumour dataset.

Supplementary Data 5: Cell per cluster, per patient, for the background and healthy dataset.

Supplementary Data 6: Results of the Wilcoxon Rank Sum and Bonferroni correction on the cell type proportion within each broad annotation cluster in the unenriched cell samples (CD235-), comparing tumour and background.

Supplementary Data 7: Results of the Wilcoxon Rank Sum and Bonferroni correction on the broad cell type abundance in the unenriched cell samples (CD235-), comparing LUAD and LUSC tumour samples.

Supplementary Data 8: Results of the Wilcoxon Rank Sum and Bonferroni correction on the cell type proportion within each broad annotation cluster in the unenriched cell samples (CD235-), comparing LUAD and LUSC tumour samples.

Supplementary Data 9: CellphoneDB results obtained on the LUAD tumour dataset.

Supplementary Data 10: CellphoneDB results obtained on the LUSC tumour dataset.

Supplementary Data 11: CellphoneDB results obtained on the background dataset.

Supplementary Data 12: CellphoneDB results obtained on the healthy dataset.

Supplementary Data 13: Results of the Wilcoxon Rank Sum and Bonferroni correction on the broad immune cell type abundance estimated by cell2location from the Visium dataset, comparing tumour and background sections.

Supplementary Data 14: Results of the Wilcoxon Rank Sum and Bonferroni correction on the non-immune cell types abundance estimated by cell2location from the Visium dataset, comparing tumour and background sections.

Supplementary Data 15: Results of the Chi2 test and Bonferroni correction on the spatial abundance estimated by Cell2location (using the 5th quantile of the abundance distribution). The Bonferroni correction was applied considering all the significant L-R pairs identified by CellphoneDB for all patients (i.e. by a factor of 309*8=2472).

Supplementary Data 16: DEA results comparing AT2 cells in tumour vs background.

Supplementary Data 17: DEA results comparing macrophages clusters and CAMLs in LUAD vs LUSC.

Supplementary Data 18: DEA results comparing alveolar macrophages (AM) in tumour vs background.

Supplementary Data 19: DEA results comparing anti-inflammatory macrophages (AIM) in tumour vs background.

Supplementary Data 20: DEA results comparing alveolar macrophages (AM) and STAB1+ macrophages (STAB1) in tumour.

Supplementary Data 21: DEA results comparing anti-inflammatory macrophages (AIM) and STAB1+ macrophages (STAB1) in tumour.

Supplementary Data 22: Details of the antibodies used for flow cytometry and IHC.

Supplementary References

1. Kim, N. *et al.* Single-cell RNA sequencing demonstrates the molecular and cellular reprogramming of metastatic lung adenocarcinoma. *Nat. Commun.* **11**, 2285 (2020).
2. Karikoski, M. *et al.* Clever-1/Stabilin-1 Controls Cancer Growth and Metastasis. *Clin. Cancer Res.* **20**, 6452–6464 (2014).
3. Collin, M. & Bigley, V. Human dendritic cell subsets: an update. *Immunology* **154**, 3–20 (2018).
4. Szabo, P. A. *et al.* Single-cell transcriptomics of human T cells reveals tissue and activation signatures in health and disease. *Nat. Commun.* **10**, 4706 (2019).
5. Gu-Trantien, C. *et al.* CXCL13-producing TFH cells link immune suppression and adaptive memory in human breast cancer. *JCI Insight* **2**, e91487, 91487 (2017).
6. Li, C., Jiang, P., Wei, S., Xu, X. & Wang, J. Regulatory T cells in tumor microenvironment: new mechanisms, potential therapeutic strategies and future prospects. *Mol. Cancer* **19**, 116 (2020).
7. Kim, N. *et al.* Single-cell RNA sequencing demonstrates the molecular and cellular reprogramming of metastatic lung adenocarcinoma. *Nat. Commun.* **11**, 2285 (2020).
8. Chan, I. S. *et al.* Cancer cells educate natural killer cells to a metastasis-promoting cell state. *J. Cell Biol.* **219**, e202001134 (2020).
9. Kerdiles, Y. M. *et al.* Natural-Killer-like B Cells Display the Phenotypic and Functional Characteristics of Conventional B Cells. *Immunity* **47**, 199–200 (2017).
10. Wang, S. *et al.* Natural Killer-like B Cells Prime Innate Lymphocytes against Microbial Infection. *Immunity* **45**, 131–144 (2016).
11. Li, Y. *et al.* GATA2 regulates mast cell identity and responsiveness to antigenic stimulation by promoting chromatin remodeling at super-enhancers. *Nat. Commun.* **12**, 494 (2021).
12. Wu, F. *et al.* Single-cell profiling of tumor heterogeneity and the microenvironment in advanced non-small cell lung cancer. *Nat. Commun.* **12**, 2540 (2021).
13. Lotfollahi, M. *et al.* Mapping single-cell data to reference atlases by transfer learning. *Nat. Biotechnol.* **40**, 121–130 (2022).
