# Supplementary material for: Single-cell and spatial transcriptomics analysis of non-small cell lung cancer
Source: Nat Commun. 2024 May 23;15:4388. doi: 10.1038/s41467-024-48700-8 (PMC11116453; doi:10.1038/s41467-024-48700-8)
Supplement: Supplementary file 3 — Reporting Summary [file 41467_2024_48700_MOESM3_ESM.pdf]

nature portfolio

Corresponding author(s): Ana Cvejic
Last updated by author(s): 30.01.2024

Reporting Summary

Nature Portfolio wishes to improve the reproducibility of the work that we publish. This form provides structure for consistency and transparency in reporting. For further information on Nature Portfolio policies, see our Editorial Policies and the Editorial Policy Checklist.

Statistics

For all statistical analyses, confirm that the following items are present in the figure legend, table legend, main text, or Methods section.

n/a	Confirmed	
☐	☒	The exact sample size (*n*) for each experimental group/condition, given as a discrete number and unit of measurement
☐	☒	A statement on whether measurements were taken from distinct samples or whether the same sample was measured repeatedly
☐	☒	The statistical test(s) used AND whether they are one- or two-sided<br>*Only common tests should be described solely by name; describe more complex techniques in the Methods section.*
☐	☒	A description of all covariates tested
☐	☒	A description of any assumptions or corrections, such as tests of normality and adjustment for multiple comparisons
☐	☒	A full description of the statistical parameters including central tendency (e.g. means) or other basic estimates (e.g. regression coefficient) AND variation (e.g. standard deviation) or associated estimates of uncertainty (e.g. confidence intervals)
☐	☒	For null hypothesis testing, the test statistic (e.g. *F*, *t*, *r*) with confidence intervals, effect sizes, degrees of freedom and *P* value noted<br>*Give P values as exact values whenever suitable.*
☒	☐	For Bayesian analysis, information on the choice of priors and Markov chain Monte Carlo settings
☒	☐	For hierarchical and complex designs, identification of the appropriate level for tests and full reporting of outcomes
☐	☒	Estimates of effect sizes (e.g. Cohen's *d*, Pearson's *r*), indicating how they were calculated

Our web collection on statistics for biologists contains articles on many of the points above.

Software and code

Policy information about availability of computer code

Data collection	CellRanger toolkit (version 3.1.0), SpaceRanger (version 1.1.0)
Data analysis	CopyKAT (version 1.0.5), CellPhoneDB, Python and R analysis environment (as indicated in Gitlab repository), Fiji (version 2.9.0)

For manuscripts utilizing custom algorithms or software that are central to the research but not yet described in published literature, software must be made available to editors and reviewers. We strongly encourage code deposition in a community repository (e.g. GitHub). See the Nature Portfolio guidelines for submitting code & software for further information.

Data

Policy information about availability of data

All manuscripts must include a data availability statement. This statement should provide the following information, where applicable:

- Accession codes, unique identifiers, or web links for publicly available datasets
- A description of any restrictions on data availability
- For clinical datasets or third party data, please ensure that the statement adheres to our policy

The scRNA-seq and Visium datasets generated in this study are available at BioStudies with accession number E-MTAB-13526 and E-MTAB-13530, respectively. The scripts used for all the analyses and to produce all the figures in the manuscript are available at https://gitlab.com/cvejic-group/lung and https://github.com/sdentro/copykat_pipeline.

Research involving human participants, their data, or biological material

Policy information about studies with human participants or human data. See also policy information about sex, gender (identity/presentation), and sexual orientation and race, ethnicity and racism.

Reporting on sex and gender	Sex was assigned (15 male and 12 female patients/donors). Sex-based analyses were not performed due to limited sample size. Gender was not determined.
Reporting on race, ethnicity, or other socially relevant groupings	n/a
Population characteristics	Please see all available data in Suppl. Table 1: Sex (15 male and 12 female), Age (37-82), Cancer type, Cancer stage, Smoking history, Location of tumour.
Recruitment	Tissue used in the research study was obtained from the Papworth Hospital Research Tissue Bank. Written consent was obtained for all tissue samples using Papworth Hospital Research Tissue Bank's ethical approval (East of England - Cambridge East Research Ethics Committee). Human tumour and adjacent background tissues, collected from the edges of the lungs, were obtained from 25 patients following tumour resection. Human healthy lung samples were obtained from two healthy deceased donors. Both healthy samples were evaluated by an expert pathologist to exclude the presence of malignancies. The human material was provided by the Royal Papworth Tissue Bank (T02229), in accordance with the HMDMC Human Tissue Act Sample Custodian Form Version 7.0 (UK NRES REC approval reference number(s): 08/H0304/56+5; HMDMC 16|094). NSCLC FFPE tumour blocks (n=2) used for validation of STAB1+ macrophages with Akoya were obtained from 2 different donors and purchased from BioIVT (ex-Asterand Bioscience). Informed Consent Form (ICF) and Institutional Review Board Approval Letter (IRBA) were obtained for all tissue samples.
Ethics oversight	1.) Royal Papworth Tissue Bank: East of England - Cambridge East Research Ethics Committee, in accordance with the HMDMC Human Tissue Act Sample Custodian Form Version 7.0 (UK NRES REC approval reference number(s): 08/H0304/56+5; HMDMC 16|094). 2.) BioIVT (ex-Asterand Bioscience): Institutional Review Board.

Note that full information on the approval of the study protocol must also be provided in the manuscript.

Field-specific reporting

Please select the one below that is the best fit for your research. If you are not sure, read the appropriate sections before making your selection.

☒ Life sciences ☐ Behavioural & social sciences ☐ Ecological, evolutionary & environmental sciences

For a reference copy of the document with all sections, see nature.com/documents/nr-reporting-summary-flat.pdf

Life sciences study design

All studies must disclose on these points even when the disclosure is negative.

Sample size	Sample size was determined by availability of patient material, balanced numbers of the two cancer types, and required cell numbers for the analyses.
Data exclusions	No data were excluded.
Replication	All attempts at replication are included in the manuscript and were successful. The number of replications for individual experiments are described in the manuscript.
Randomization	Not applicable (study based on computational data analysis). Cancer type and stage or absence of malignancies, respectively, were evaluated by expert pathologists (Papworth Hospital Research Tissue Bank) before analysis of the samples.
Blinding	Blinding was not relevant, as the study is based on computational data analysis and does not involve subjective assessments.

Reporting for specific materials, systems and methods

We require information from authors about some types of materials, experimental systems and methods used in many studies. Here, indicate whether each material, system or method listed is relevant to your study. If you are not sure if a list item applies to your research, read the appropriate section before selecting a response.

Materials & experimental systems

n/a	Involved in the study
	[x] Antibodies
[x]	Eukaryotic cell lines
[x]	Palaeontology and archaeology
[x]	Animals and other organisms
[x]	Clinical data
[x]	Dual use research of concern
[x]	Plants

Methods

n/a	Involved in the study
[x]	ChIP-seq
	[x] Flow cytometry
[x]	MRI-based neuroimaging

Antibodies

Antibodies used

CD3 (Biolegend, 317340), CD19 (Biolegend 302226), CD56 (BD Biosciences 557919), CD45 (ThermoFisher MHCD4501), CD33 (Biolegend 303406), HLA_DR (Biolegend 307635), CD68 (Abcam AB213363), STAB1 (SantaCruz SC293254). Compare Suppl. Table 22.

Validation

All antibodies are commercially available and have previously been used for the respective purposes using human cells. The relevant data and publications can be found on the manufacturer's websites (links are included in Suppl. Table 22).

Plants

Seed stocks

Report on the source of all seed stocks or other plant material used. If applicable, state the seed stock centre and catalogue number. If plant specimens were collected from the field, describe the collection location, date and sampling procedures.

Novel plant genotypes

Describe the methods by which all novel plant genotypes were produced. This includes those generated by transgenic approaches, gene editing, chemical/radiation-based mutagenesis and hybridization. For transgenic lines, describe the transformation method, the number of independent lines analyzed and the generation upon which experiments were performed. For gene-edited lines, describe the editor used, the endogenous sequence targeted for editing, the targeting guide RNA sequence (if applicable) and how the editor was applied.

Authentication

Describe any authentication procedures for each seed stock used or novel genotype generated. Describe any experiments used to assess the effect of a mutation and, where applicable, how potential secondary effects (e.g. second site T-DNA insertions, mosiacism, off-target gene editing) were examined.

Flow Cytometry

Plots

Confirm that:

- [x] The axis labels state the marker and fluorochrome used (e.g. CD4-FITC).
- [x] The axis scales are clearly visible. Include numbers along axes only for bottom left plot of group (a 'group' is an analysis of identical markers).
- [x] All plots are contour plots with outliers or pseudocolor plots.
- [x] A numerical value for number of cells or percentage (with statistics) is provided.

Methodology

Sample preparation

On the day of FACS sorting, cells were rapidly thawed at 37 degrees and transferred to complete RPMI media. Live cell enrichment was performed using MACS Dead Cell Removal Kit (Miltenyi Biotec) following the manufacturer's instructions. Red blood cells were further depleted by negative selection using CD235a Microbeads (Miltenyi Biotec) and MACS LS columns (Miltenyi Biotec), following the manufacturer's instructions.
For FACS sorting, cells were stained with Zombie Aqua to exclude dead cells and the cocktail of antibodies for 30 minutes at 4 °C. Cells were centrifuged for 5 minutes at 300 g, 4 °C, resuspended in 500 µl of 5% FBS in PBS and subsequently filtered into polypropylene FACS tubes.
Immune cells were sorted as live, CD45+; MDSC were sorted as live, CD45+, Lineage- (Lin: CD3, CD56, CD19), CD33+, HLA-DR-/low (Supp. Table 22, Supp. Figure 1A). Cells were sorted into a 1.5 ml tube, counted and submitted for 10x scRNA seq library preparation.

Instrument

Cell were sorted at the Cambridge Biomedical Research Centre Flow cytometry Core facility on BD Aria II, BD Aria III, and BD Influx sorters.

Software

Collection: BD FACSDiva, Analysis: FlowJo

Cell population abundance

The relevant cell population abundance is shown in Supplementary Figure 1. The identity and purity of the sorted cells was

	further confirmed by scRNA-seq.
Gating strategy	Gating strategy used to enrich CD45+ and MDSCs: Debris was excluded by gating on FSC-A/SSC-A; singlets were gated on FCS/TPW; dead cells were excluded by gating on Zombie-aqua negative cells, then CD45+ cells were sorted. MDSCs were identified as CD45 positive, Lineage (CD3, CD19, CD56) negative, CD33 positive, HLA-DR dim/negative cells.

☒ Tick this box to confirm that a figure exemplifying the gating strategy is provided in the Supplementary Information.
