# Supplementary material for: Single-cell and spatial transcriptomics analysis of non-small cell lung cancer
Source: Nat Commun. 2024 May 23;15:4388. doi: 10.1038/s41467-024-48700-8 (PMC11116453; doi:10.1038/s41467-024-48700-8)
Supplement: Supplementary file 4 — Description of Additional Supplementary Files [file 41467_2024_48700_MOESM4_ESM.pdf]

Description of Additional Supplementary Files

File Name: Supplementary Data 1

Description:

STab1: Sample metadata, including scRNA-seq metrics, spatial transcriptomics metrics, and available clinical data.

STab2: Results of the Wilcoxon Rank Sum and Bonferroni correction on the non-immune cell type abundance in the unenriched cell samples (CD235-), comparing tumour and background.

STab3: Results of the Wilcoxon Rank Sum and Bonferroni correction on the broad immune cell type abundance in the unenriched cell samples (CD235-), comparing tumour and background.

STab4: Cell per cluster, per patient, for the tumour dataset.

STab5: Cell per cluster, per patient, for the background and healthy dataset.

STab6: Results of the Wilcoxon Rank Sum and Bonferroni correction on the cell type proportion within each broad annotation cluster in the unenriched cell samples (CD235-), comparing tumour and background.

STab7: Results of the Wilcoxon Rank Sum and Bonferroni correction on the broad cell type abundance in the unenriched cell samples (CD235-), comparing LUAD and LUSC tumour samples.

STab8: Results of the Wilcoxon Rank Sum and Bonferroni correction on the cell type proportion within each broad annotation cluster in the unenriched cell samples (CD235-), comparing LUAD and LUSC tumour samples.

STab9: CellphoneDB results obtained on the LUAD tumour dataset.

STab10: CellphoneDB results obtained on the LUSC tumour dataset.

STab11: CellphoneDB results obtained on the background dataset.

STab12: CellphoneDB results obtained on the healthy dataset.

STab13: Results of the Wilcoxon Rank Sum and Bonferroni correction on the broad immune cell type abundance estimated by cell2location from the Visium dataset, comparing tumour and background sections.

STab14: Results of the Wilcoxon Rank Sum and Bonferroni correction on the non-immune cell types abundance estimated by cell2location from the Visium dataset, comparing tumour and background sections.

STab15: Results of the Chi2 test and Bonferroni correction on the spatial abundance

estimated by Cell2location (using the 5th quantile of the abundance distribution). The Bonferroni correction was applied

considering all the significant L-R pairs identified by CellphoneDB for all patients (i.e. by a factor of 309*8=2472).

STab16: DEA results comparing AT2 cells in tumour vs background.

STab17: DEA results comparing macrophages clusters and CAMLs in LUAD vs LUSC.

STab18: DEA results comparing alveolar macrophages (AM) in tumour vs background.

STab19: DEA results comparing anti-inflammatory macrophages (AIM) in tumour vs background.

STab20: DEA results comparing alveolar macrophages (AM) and STAB1+ macrophages (STAB1) in tumour.

STab21: DEA results comparing anti-inflammatory macrophages (AIM) and STAB1+ macrophages (STAB1) in tumour.

STab22: Details of the antibodies used for flow cytometry and IHC.
